# An ensemble machine learning approach for predicting anemia among under-five children in malaria-endemic sub-Saharan African countries

**DOI:** 10.1186/s40249-026-01461-6

**Published:** 2026-07-13

**Authors:** Berhan Tekeba, Nebebe Demis Baykemagn, Alexander Takele Mengesha, Melaku Alelign Mengstie

**Affiliations:** 1 Department of Pediatrics and Child Health Nursing, School of Nursing, College of Medicine and Health Sciences, University of Gondar, Gondar, Ethiopia; 2 Department of Health Informatics, Institute of Public Health, College of Medicine and Health Sciences, University of Gondar, Gondar, Ethiopia; 3 Department of Information Science, College of Informatics, University of Gondar, Gondar, Ethiopia

**Keywords:** Anemia, Malaria, Sub-Saharan Africa, Malaria endemic, Machine-learning, Predictors

## Abstract

**Background:**

Worldwide, anemia in children under-five is a major public health issue, particularly in sub-Saharan Africa. Sub-Saharan Africa also has the highest burden of malaria. This study aimed to develop an ensemble machine learning model to estimate anemia burden and potential predictors in under-five children in malaria-endemic sub-Saharan African countries.

**Method:**

A cross-sectional study was conducted using Demographic and Health Survey data from sub-Saharan African countries. Samples were selected through a two-stage stratified cluster sampling method. Data analysis was performed using Python 3.8, with a total weighted sample of 21,249. The dataset was split into 80% for training and 20% for testing and validation purposes. To address class imbalance, a hybrid data balancing approach combining SMOTE (Synthetic Minority Over-sampling Technique) and Tomek Links was applied. Four machine learning algorithms were developed and evaluated using standard performance metrics. Recursive Feature Elimination with a Random Forest classifier was used to identify potential predictors of anemia among children under five living in malaria-endemic SSA countries.

**Result:**

In this study, XGBoost showed the best performance, achieving an accuracy of 83.69%, a precision of 85.81%, and an F1 score of 83.19%. Additionally, XGBoost attained the highest ROC AUC of 90.1 and Precision Recall AUC of 90.0. According to Recursive Feature Elimination with a Random Forest classifier, region, birth order, child age, wealth index, and number of mosquito nets were identified as the associated factors of anemia among under-five children in malaria-endemic SSA countries.

**Conclusion:**

To reduce anemia among under-five children in malaria-endemic regions of sub-Saharan Africa, interventions should prioritize implementing geographically targeted programs, focus on younger children and those with high birth orders by integrating anemia screening into routine check-ups. In addition, enhancing economic support for low-income families and distributing and educating families on the proper use of mosquito nets are essential.

**Supplementary Information:**

The online version contains supplementary material available at 10.1186/s40249-026-01461-6.

## Background

Anemia is a condition characterized by a reduced quantity of red blood cells, or hemoglobin, which transports oxygen to bodily tissues [1]. A hemoglobin content of less than 11 g/dl is considered anemia by the World Health Organization (WHO) in children under the age of five years [2]. Malaria infection is the leading cause of morbidity and mortality in children under five in sub-Saharan African countries [3]. Anemia is one of the major complications of malaria infection [4]. There is evidence in research that anemia and malaria interrelate, resulting in adverse health outcomes and mortality, especially in children under the age of five [22]. Malaria infection causes hemolysis of parasitized and non-parasitized erythrocytes and bone marrow dys-erythropoiesis that compromise rapid recovery from anemia [5]. Anemia also contributes directly or indirectly to a significant proportion of malaria-related deaths [6, 7].

Worldwide, anemia in children under-five is a major public health issue. According to statistics from the World Health Organization, 40% of children under the age of five were anemic [8, 9]. The prevalence varies across countries, with the highest percent observed in sub-Saharan Africa (SSA) (60.2%) and South Asia [10]. In 2019, globally, the prevalence of anemia in children aged 6 to 59 months was 39.8%, with the highest rate in the African Region at 60.2% [11].

Anemia has multiple causes, with infectious illness and nutritional deficiency being the primary causes in Sub-Saharan Africa (SSA) [12, 13]. Other reasons include hemolysis due to congenital or acquired blood disorders, blood loss, hemoglobinopathies, and infections such as malaria, tuberculosis, and HIV [14]. Previous studies have identified several determinants [15–21]. However, most traditional regression approaches assume linear relationships and are limited by multicollinearity and the number of risk factors. These limitations reduce their ability to capture complex interactions common in malaria-endemic settings.

Machine learning (ML) methods can overcome these challenges by accommodating large numbers of potential predictors, handling nonlinear relationships, and improving classification performance [22]. Yet, despite the high burden of anemia in malaria-endemic areas [23], few studies have deployed an ML to identify the most important determinants systematically [24]. Furthermore, single machine learning models may be limited by algorithm-specific bias and sensitivity to data structure. Ensemble learning methods address these limitations by combining multiple learners, thereby improving predictive accuracy, stability, and generalizability [25].

To achieve the targets of reducing child mortality of the Sustainable Development Goal (SDG)−2030, it is necessary to generate adequate evidence on the burden of anemia and its determinants, which is highly crucial for the development of timely interventions in anemia prevention and treatment that will help policymakers to design more precise, region-specific interventions targeting potential risk factors. Despite this, there is a paucity of up-to-date information on the potential predictors of anemia in malaria-endemic areas. Most of the prior studies utilized traditional logistic regression to determine the magnitude and associated factors of anemia in malaria-endemic areas. To address this gap, this study aimed to identify key associated factors of anemia among children under five living in malaria-endemic regions by deploying a machine approach to generate evidence for informing targeted interventions.

## Method

### Data source

The dataset we used in this study was obtained from the Demographic and Health Survey Data (DHS) after a formal request. The DHS Program is a global data-gathering project that offers detailed and high-quality information about population demographics, health, and nutrition. DHS surveys, mostly funded by the United States Agency for International Development (USAID), are undertaken in low- and middle-income countries around the world to help governments, researchers, and organizations monitor and improve public health policies and social services. We have included 17 sub-Saharan African countries that had recent DHS data from 2015 to 2024 to inform relevant authorities with up-to-date information. Burkina Faso, Cameroon, Ghana, Guinea, Kenya, Liberia, Madagascar, Mali, Malawi, Mozambique, Nigeria, Niger, Sierra Leone, Senegal, Togo, Tanzania, and Uganda. The DHS paternal (PR) file data set was used, which had records for every child of interviewed women, born in the two years preceding the survey. The data set from the above countries was appended together using Python software to determine the predictors of anemia among children under five in malaria-endemic sub-Saharan African countries.

### Sampling procedure

The DHS surveys use a stratified, two-stage cluster sampling technique. The first step was the random selection of clusters, or enumeration areas (EAs), that encompass the entire country from the sampling frame derived from the most recent national survey that was made accessible. EAs are small, well-defined geographic areas often delineated as a part of a national census that serve as a building block for the national survey, which is used as a sampling unit. Each EA is designed to include a manageable number of households. In the second step, interviews were held in a subset of the target population's households using systematic sampling, which was applied to all of the households mentioned in each cluster (women aged 15–49). A total weighted sample of 23,025 woman-child pairs, 2 years preceding the survey, was included in the study. For households that had more than one child in the 2 years before the survey, the youngest child was included in the study.

#### Outcome of the variable

The outcome variable is anemia among children under five. According to the WHO criteria, children aged 6–59 months are considered anemic if their hemoglobin is less than 11 g/dl. The outcome was coded as “Yes = 1” for anemic children and “No = 0” for non-anemic children.

#### Independent variables

Both individual and community-level factors were reviewed from different literatures, and these include child age, maternal education, wealth index, birth order, postnatal care (PNC), and source of drinking water [16, 26–31].

### Data management and analysis

#### Data acquisition

The dataset was downloaded in dta extension format, and the data cleaning and labeling were done. Python 3.8, particularly Jupyter Notebook in Anaconda Navigator, was used for data management and model building. Important libraries such as Pandas, NumPy, and Seaborn were imported from Python. Python packages such as Metaplot-lib and sklearn were used to import important supervised machine learning algorithms for data analysis and visualization. An Ensemble machine learning algorithm, Random Forest, XGBoost, Cat Boost, and LightGBM are trained with hyperparameters optimized through grid search. Model performance is evaluated on the test set using accuracy, precision, recall, F1-score, and AUC-ROC metrics. The best-performing model is selected primarily based on accuracy and precision. Upon satisfactory performance, the model is employed for anemia prediction.

#### Data preprocessing

After obtaining the secondary dataset from DHS, the data preprocessing phase involves several key steps: data integration, cleaning, transformation, feature selection, class balancing, data splitting, and other necessary preparations to ensure data quality and suitability for modeling.

### 1. Data cleaning

#### Handling missing values

Missing values were addressed according to the nature of each feature to maintain data integrity. For categorical variables such as residence, source of drinking water, and toilet facility, missing entries were imputed using the mode, the most frequently occurring value within each feature. For continuous numerical variables like the number of household members, child age, and the number of mosquito nets, missing values were filled with the mean of the available data. This strategy preserved the statistical distribution of the dataset and ensured balanced and reliable input for further analysis.

#### Handling redundancy

During data preprocessing, 1776 duplicate records were detected using the duplicated () function. These duplicates, possibly resulting from data collection or merging errors, were removed to maintain data quality. Eliminating duplicates helped prevent bias and over-fitting, ensuring the dataset contained unique observations for reliable model training and evaluation.

### 2. Data transformation

Several continuous and categorical features were transformed into meaningful categories. Continuous variables, such as the number of household members (hv014) and the number of children under five (hv009), were binned into ordered categories using predefined thresholds informed by their distributions and relevant literature. Cluster altitude (hv040) was grouped into ranges to account for non-linear associations with child health outcomes. Multi-category features, including source of drinking water, toilet facility, main floor material, wall material, main roof material, type of cooking fuel, and whether children sleep under a net, were recoded into binary indicators by combining categories associated with similar risk or protective effects. High-cardinality variables, such as child age and birth order, were also binned into fewer, interpretable levels. These binning and recoding steps reduced dimensionality, enhanced feature interpretability, and improved model stability.

To assess the robustness of these transformations, sensitivity analyses were conducted by testing alternative binning schemes for key variables. Model performance metrics (accuracy, AUC) and feature importance rankings remained consistent across alternative categorizations, confirming that the selected binning and recoding choices did not materially affect predictive outcomes.

#### Feature selection

To identify the most relevant features for the prediction task, we employed Recursive Feature Elimination using a Random Forest classifier as the base estimator. This method iteratively removed less important features based on the model’s internal feature importance scores. After evaluating performance across different subset sizes, we selected 22 optimal number of features based on accuracy metrics. The final RFE model retained the most relevant features, enhancing model performance by reducing dimensionality, minimizing noise, and focusing the learning process on attributes most predictive of the target outcome.

### A. Data splitting

The original dataset consisted of 23,025 records and 29 features. After data cleaning, which involved handling missing values, removing duplicates, and dropping irrelevant features, the dataset was reduced to 21,249 records and 27 features. The cleaned data was then split into training and testing subsets using an 80 to 20 ratio through the train-test-split function. Stratified sampling was applied to ensure the class distribution remained consistent across both sets, resulting in balanced x-train, x-test, y-train, and y-test datasets for model training and evaluation.

### B. Addressing class imbalance

**Fig. 1:**
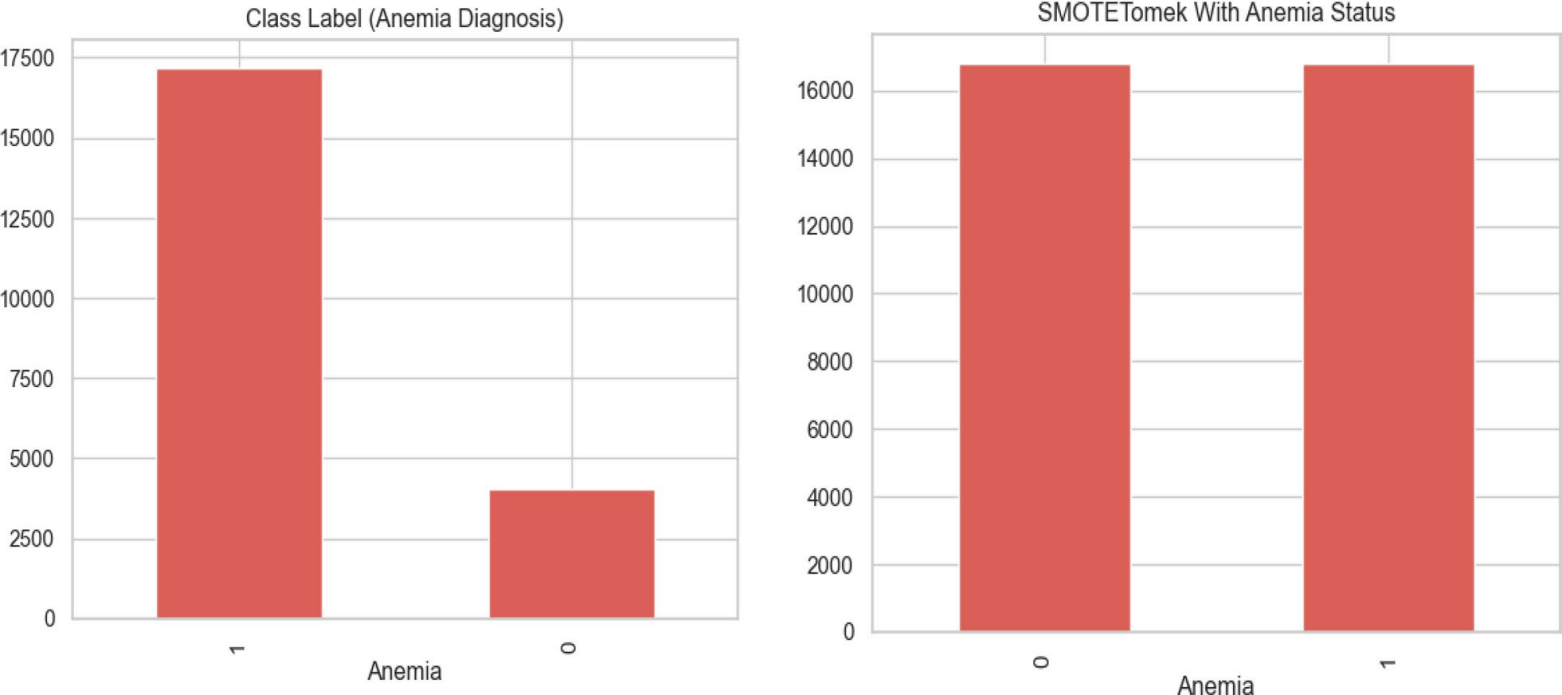
Class label before and after applying SMOTE-Tomek

The target feature, Anemia, is imbalanced, with 17,193 cases (80.91%) labeled as anemic (class 1) and 4,056 cases (19.09%) labeled as non-anemic (class 0). This imbalance underscores the need for class balancing methods to avoid bias in model learning, particularly favoring the majority class. The skewed class distribution was visually confirmed using a count plot, indicating a potential tendency for the model to predict the majority class more often. To address this, we applied the SMOTE-Tomek technique exclusively to the training data. SMOTE (Synthetic Minority Over-sampling Technique) increases the representation of the minority class by generating synthetic examples, while Tomek Links helps clean the data by removing overlapping samples near class boundaries. As a result, the training dataset was balanced to 13,436 cases for each class, as shown in Fig. 1. This hybrid resampling approach improved the model’s ability to generalize without introducing artificial balance into the test data.

### C. Feature selection

We employed Recursive Feature Elimination (RFE) using a Random Forest classifier as the base estimator for identifying relevant features. The method iteratively removed less important features based on the model’s internal feature importance scores. After evaluating model performance across different subset sizes, we selected the optimal number of features, 22 in this case, based on accuracy metrics. The final RFE model retained the most informative features, enhancing model performance by reducing dimensionality, minimizing noise, and focusing the learning process on the attributes most predictive of the target outcome.

### D. Model building and evaluation

Ensemble machine learning algorithms, Random Forest, XGBoost, CatBoost, and LightGBM, are trained with hyperparameters optimized through grid search. Model performance is evaluated on the test set using accuracy, precision, recall, F1-score, and AUC-ROC metrics. The best-performing model is selected primarily based on accuracy and precision. Upon satisfactory performance, the model is employed for anemia prediction. Additionally, explainable AI methods, particularly SHAP analysis, were employed to provide transparency and offer insights into the factors influencing the model's predictions.

## Results

### Socio-demographic characteristics

A total of 21,249 children under the age of five were included in the study. Nearly half (48.40%) of households had 3 to 4 members. The vast majority (85.78%) of study participants resided in rural areas. About 39.47% of participants had an unimproved source of drinking water. More than two-thirds (68.22%) of households lacked improved toilet facilities. Over half (55.84%) of households did not own a radio, and only 15.92% had access to television. The majority (81.48%) of households were headed by males. More than three-fourths (78.15%) of households had two or more rooms for sleeping. Nearly two-thirds (64.99%) of children slept under treated bed nets. Regarding wealth status, over 60% of households fell within the poorest and poor categories. Most children (77.06%) were aged between 24 and 59 months (Table 1).

**Table 1:** Socio-demographic characteristics of study subjects

Variable	Category	Frequency	Percentage (%)
Number of household members	1–2	6,753	31.78%
	3–4	10,287	48.40%
	≥ 5	4,209	19.82%
Number of under-five children	0	13,138	61.84%
	1	6,252	29.42%
	2 or more	1,859	8.75%
Residence	Rural	18,226	85.78%
	Urban	3,023	14.22%
Source of drinking water	Improved	12,864	60.53%
	Unimproved	8,385	39.47%
Toilet facility	Unimproved	14,495	68.22%
	Improved	6,754	31.78%
Radio	No	11,865	55.84%
	Yes	9,384	44.16%
TV	No	17,869	84.08%
	Yes	3,380	15.92%
Main floor material	Unimproved	13,403	63.05%
	Improved	7,846	36.95%
Wall material	Unimproved	15,126	71.14%
	Improved	6,123	28.86%
Main roof material	Improved	14,243	67.01%
	Unimproved	7,006	32.99%
Room for sleeping	1	4,641	21.85%
	2	6,651	31.30%
	3 +	9,957	46.85%
Sex of household head	Male	17,307	81.48%
	Female	3,942	18.52%
Children sleep under bed net	Yes	13,806	64.99%
	No	7,443	35.01%
Water source location (hv235)	In yard	18,260	85.93%
	Out of yard	2,989	14.07%
Mobile phone	Yes	15,863	74.66%
	No	5,386	25.34%
Wealth index	Poorest	6,971	32.81%
	Poor	5,963	28.07%
	Middle	4,604	21.67%
	Rich	2,814	13.24%
	Richest	897	4.22%
Number of mosquito nets	0	4,617	21.73%
	1	3,561	16.76%
	2	4,576	21.53%
	3 +	8,495	39.98%
Child age (months)	0–5	44	0.21%
	6–11	1,149	5.41%
	12–23	3,683	17.33%
	24–59	16,373	77.06%
Child sex	Male	10,995	51.74%
	Female	10,254	48.26%
Mother's education	No education	13,472	63.39%
	Primary	4,955	23.32%
	Secondary and above	2,822	13.29%
Birth order	First	12,141	57.14%
	Second–Fourth	6,051	28.47%
	Fifth and above	3,057	14.39%
Region	Central	2,783	13.10%
	East	3,828	18.02%
	West	14,638	68.88%

### Important attributes of anemia under five in malaria-endemic areas

Multicollinearity among candidate predictors was first assessed using a correlation matrix as presented in (the supplementary Fig. 1). Pairwise correlation analysis (|r|> 0.8) indicated that no variables exhibited problematic correlations, and therefore no features were removed at this stage. All candidate predictors were consequently retained for the feature selection process (Supplementary Fig. 1).

Subsequently, Recursive Feature Elimination was applied using a Random Forest classifier as the base estimator. A range of feature subset sizes was evaluated by iteratively fitting the RFE model with varying numbers of retained features. At each iteration, model performance was assessed using test set accuracy to determine the optimal feature subset. Based on this evaluation, 22 features were identified as the optimal set.

The final RFE model was then trained using the Random Forest classifier, retaining the top 22 most informative features. The selected predictors encompassed key household, demographic, socioeconomic, and child-related variables, including wealth index, household composition, sleeping arrangements, regional factors, mother’s education, birth order, access to media and utilities, water source, sanitation, and child characteristics. This feature selection process enhanced model performance by focusing learning on the most predictive attributes while reducing noise and dimensionality.

As presented in supplementary Fig. 2, the recursive ffeature elimination with a Random Forest estimator identified 22 features as the most informative predictors of anemia among children under five. These included wealth index, household composition, sleeping arrangements, regional factors, mother’s education, birth order, access to media and utilities, water source, sanitation, and child characteristics (Supplementary Fig. 2).

### Model performance to predict anemia among under-fives in malaria-endemic sub-Saharan African countries

Model performance was evaluated using multiple classification metrics, including accuracy, precision, recall, F1 score, and ROC–AUC (Table 2), to provide a comprehensive assessment of predictive capability. While accuracy was reported to facilitate comparison across models, it was not used as the sole criterion for model selection because it may obscure clinically meaningful errors in health prediction tasks.

**Table 2:** Performance of ensemble machine learning algorithms

Model	Accuracy	Precision	Recall	F1 Score
Random Forest	82.45	83.05	81.56	82.30
CatBoost	80.16	81.61	77.88	79.70
XGBoost	83.69	85.81	80.73	83.19
LightGBM	82.35	84.67	79.01	81.74

In the context of childhood anemia, recall (sensitivity) is particularly important, as failing to identify anemic children (false negatives) can delay intervention and increase the risk of adverse health outcomes. Precision was also considered to ensure that identified cases were reliable, thereby minimizing unnecessary follow-up. The F1 score was used to capture the balance between precision and recall, especially given the heterogeneous distribution of anemia risk factors across malaria-endemic sub-Saharan African countries. Additionally, ROC–AUC was employed to evaluate the models’ discriminative ability across varying classification thresholds.

Among the four evaluated ensemble algorithms, Random Forest, CatBoost, XGBoost, and LightGBM, XGBoost demonstrated the most favourable trade-off across these prioritized metrics, achieving the highest accuracy (83.69%), precision (85.81%), and F1 score (83.19%), while maintaining a strong recall of 80.73%. Although Random Forest and LightGBM showed competitive performance, XGBoost’s superior balance between precision and recall indicates its greater effectiveness in identifying children at risk of anemia while limiting misclassification, making it the most suitable model for this prediction task as presented in Table 2 and Supplementary Fig. 3.

Generally, XGBoost outperformed Random Forest, CatBoost, and LightGBM in predicting anemia among children under five due to its ability to capture complex, non-linear relationships and interactions among diverse household, socioeconomic, and child-related predictors. Its gradient boosting framework builds trees sequentially, with each tree correcting errors from the previous ones, while built-in L1 and L2 regularization prevent overfitting even in the presence of correlated features. XGBoost’s efficient handling of missing values, heterogeneous data, and optimized gradient-based learning further enhances predictive performance. Additionally, the model effectively weights features based on their contribution to reducing error, allowing it to focus on the most informative attributes and achieve a superior balance of recall and precision. In comparison, Random Forest grows trees independently without sequential correction, CatBoost requires careful tuning for categorical variables, and LightGBM may be sensitive to class imbalance, making XGBoost the most robust and accurate choice for identifying children at risk of anemia across malaria-endemic sub-Saharan African countries.

Table 2**:** Performance metrics (Accuracy, Precision, Recall, F1 Score) of four ensemble machine learning algorithms in predicting anemia among children under five in malaria-endemic sub-Saharan African countries.

As presented in supplementary Fig. 3, Receiver Operating Characteristic (ROC) and Precision-Recall (PR) curves for four machine learning models predicting anemia among children under five in malaria-endemic SSA countries. XGBoost achieved the highest performance with a ROC AUC of 90.1 and a PR AUC of 90.0, followed by Random Forest, LightGBM, and CatBoost.

### Explainable artificial intelligence analysis

Explainable AI (XAI) techniques were applied to the trained XGBoost model to quantify feature contributions to anemia predictions. SHAP (SHapley Additive exPlanations) values were computed to assess the influence of each predictor on the model output (Supplementary Fig. 4).

Supplementary Fig. 4 presents the global SHAP summary plot, showing mean absolute SHAP values for all features, which indicates their relative importance in the model. Supplementary Fig. 5 illustrates a local SHAP waterfall plot for a single instance, displaying how individual feature values contribute to that specific prediction. All visualizations provide transparency of the model’s decision-making process. Interpretation of these findings is provided in the “Discussion” section (Supplementary Fig. 5).

## Discussion

This study employed an ensemble machine learning approach to identify factors associated with anemia among under-five children living in malaria-endemic sub-Saharan African countries using nationally representative DHS data from 17 countries.

The findings of this study demonstrated the potential of ML algorithms in predicting associated factors of anemia among children under five in malaria-endemic SSA. This opens up opportunities for the development of potential automated screening tools and decision support systems that can assist health care providers for identifying potential predictors of anemia in malaria endemic SSA countries. We have utilized four different ML algorithms, Random Forest, CAT Boost, XGBoost, and Light Gradient Boost, to assess and compare their predictive capabilities. All four algorithms achieved ROC values above the optimal threshold; however, XGBoost demonstrated the best overall performance with the highest accuracy of 83.69%, precision of 85.81%, recall of 80.73%, and F1 score of 83.19%. Although Random Forest showed solid results with an accuracy of 72.3% and an AUC value of 57.8%, slight variations in metric values across studies can be attributed to differences in socio-economic factors, dataset size, and study areas. The consistent findings across studies may be due to the nature of features included, as Random Forest excels with categorical variables, high-dimensional data, and non-linear trends while requiring minimal hyperparameter tuning [82]. The use of the XGBoost classifier for identifying the potential predictors of anemia offers significant implications by providing highly accurate predictive models, uncovering key risk factors and underlying mechanisms, identifying vulnerable subgroups, and enabling the integration of machine learning into healthcare systems. These advantages pave the way for targeted interventions, personalized healthcare strategies, and improved health outcomes for individuals affected by anemia.

The findings suggest that ensemble machine learning models, particularly XGBoost, could be operationalized as decision-support tools within routine child health and malaria control programs in sub-Saharan Africa. Integrated into primary healthcare services, community health worker workflows, or digital health platforms, such models could use routinely collected variables (e.g., child age, household wealth proxies, region, birth order, and mosquito net availability) to identify under-five children at high risk of anemia for targeted screening and preventive interventions. Implementation is technically feasible since most predictors are already captured through routine health and malaria programs; however, challenges related to data quality, digital infrastructure, workforce capacity, and contextual heterogeneity remain. Effective deployment would require local model calibration, prospective validation, and alignment with national child health guidelines. Ethical and equity considerations are essential, as ML-based targeting may reinforce existing socioeconomic or geographic disparities if applied without safeguards. Therefore, such tools should complement not replace clinical judgment and universal child health strategies. The use of explainability methods, such as SHAP, supports transparency and trust, while future applications should include fairness assessments and continuous monitoring to ensure equitable benefits.

Another aim of this study was to identify associated factors of anemia among under-five children. To accomplish this, the author utilized Recursive Feature Elimination using a Random Forest classifier to select important variables. Out of a total of 22 variables included based on the literatures; the study identified the top five associated factors of anemia among under-five children. The SHAP summary plot of best best-performed algorithm revealed that region, birth order, child age, wealth index, and number of mosquito nets were found to be the top five associated factors of anemia among under-five children in malaria-endemic SSA countries.

The wealth index is significantly associated with under-five anemia. This is supported by a study done in Ethiopia [24], Rwanda [32]. The possible explanation could be that households in lower wealth categories often have limited access to iron-rich foods and nutritious diets, face greater challenges accessing healthcare services, and are more exposed to environmental factors like poor sanitation and indoor air pollution from cooking fuel [24]. The substantial link between household wealth and childhood anemia emphasizes the need for policies that go beyond clinical therapy. It is critical to improve low-income households' access to inexpensive, nutrient-dense meals, strengthen social protection systems, and expand community-based nutrition and anemia screening programs. Targeting poverty-related barriers such as inadequate sanitation, restricted healthcare access, and reliance on dangerous cooking fuels could significantly reduce anemia risk among the most disadvantaged children.

Region in SSA is one of the associated factors of anemia among under-five children in malaria-endemic SSA [33]. Some SSA parts, including West Africa, like Nigeria, Burkina Faso, and Mali, and East Africa, including South Sudan, Ethiopia, and Uganda, are high malaria endemic areas where a higher load of anemia is inevitable. Malaria causes anemia primarily by destroying red blood cells as the parasite multiplies inside them, leading to a reduced oxygen-carrying capacity in the body [34]. Additionally, the immune system may mistakenly attack both infected and healthy red blood cells, while the infection itself can suppress bone marrow activity, slowing down new red blood cell production [7]. In malaria-endemic regions, especially among under-five children, repeated infections and poor nutritional status make anemia more severe and persistent [35]. Regional differences in anemia are a reflection of larger systemic disparities in healthcare access, socioeconomic status, and malaria transmission. By increasing investment in high-transmission areas and bolstering cross-border malaria control, policies must take these geographical variations into consideration. In the worst impacted areas, combining malaria treatments with community-level surveillance, maternal-child health care, and nutrition support can help break the cycle of recurrent infections and chronic anemia.

In the SHAP summary plot, birth order is identified as the associated factor of anemia among under-five children. This is supported by other studies [36, 37]. The possible explanation might be the distribution of scarce resources within the family, and related to maternal exhaustion of micronutrients. In addition, an increase in the number of children is associated with increased health problems due to competition for food, infections, and cross-contamination [38]. Higher birth order as a predictor of anemia suggests the need to support large households through targeted nutrition, maternal health, and family-planning services to reduce resource strain and improve child health outcomes.

Child age is identified as a potential predictor of under-five anemia. This is supported by other studies [39, 40]. Children between 24 and 59 months are more vulnerable to infection due to the natural decline of maternally transferred IgG antibodies, while their immune systems are still developing through repeated exposure. Additionally, since mothers tend to sleep with and closely protect the youngest child, those children are better shielded from mosquito bites compared to older siblings. Finally, kids in the 24–59-month age group spend more time playing outdoors, increasing their likelihood of mosquito exposure [41, 42]. This age-specific susceptibility highlights a flaw in current child health initiatives, which frequently prioritize newborns while ignoring preschool-aged children, who are more susceptible to mosquito bites and have lower passive immunity. Policies should strengthen age-targeted malaria prevention, such as ensuring that nets and indoor residual spraying help older children, and expand community-based nutrition and malaria surveillance to detect anemia early in this group.

The number of available mosquito nets is also associated with under-five anemia. This is in line with other studies [43]. This is due to the fact that the number of mosquito nets in a household is a strong proxy for malaria. Insecticide-treated nets (ITNs) reduce contact with malaria-carrying mosquitoes, by which malaria is a major cause of anemia among under-five children. Mosquito net availability as a predictor of anemia highlights ongoing inadequacies in malaria prevention coverage. Policies should prioritize equitable ITN distribution, replace worn-out nets, and enhance behavioral interventions that promote proper and consistent usage. Integrating ITN distribution with child health and nutrition initiatives could give a more holistic strategy to reduce malaria-related anemia.

### Strengths and limitations

The strength of this study stemmed from its focus on several nations with varying social, cultural, and economic backgrounds that may help programmers and policymakers to create effective multi-country initiatives. The cross-sectional study design made it impossible for the study's findings to establish a causal link between the independent variables and the outcome. Recall bias may also exist because DHS is a cross-sectional survey that relies on respondents' self-reports. Due to machine learning use for analysis, the absence of regression coefficients for each predictor makes it challenging to quantify the strength of their association with anemia, which might hinder the ability to precisely measure the impact of individual predictors on the outcome.

Furthermore, this study did not explicitly incorporate DHS sampling weights, clustering, or stratification variables into the model training process, as the primary focus was on individual-level prediction of anemia rather than design-based population inference. Consequently, the models may be influenced by over-represented subpopulations and correlated observations, which could limit the generalizability of findings to population-level estimates. Future research should explore methods that integrate complex survey design features into machine learning frameworks to improve representativeness and reduce potential bias.

## Conclusion

This study demonstrates the value of ensemble machine learning methods for identifying the potential predictors of anemia among under-five children in malaria-endemic sub-Saharan African countries using large, multi-country DHS data. By leveraging the complementary strengths of multiple ensemble algorithms and explainable AI techniques, the study not only achieved high predictive performance but also identified and ranked key risk factors associated with anemia that may be difficult to capture using traditional regression approaches. Region, birth order, child age, household wealth, and the number of mosquito nets emerged as the most common risk factors, highlighting the complex interplay between geographic, socioeconomic, demographic, and malaria-related factors.

From a programmatic and policy perspective, these findings support the need for geographically targeted anemia and malaria control strategies, particularly in high-burden regions. Integrating routine anemia screening with malaria prevention services, prioritizing older preschool-aged children and those from large households, strengthening social protection and nutrition support for economically disadvantaged families, and ensuring equitable distribution and consistent use of insecticide-treated mosquito nets are critical interventions. The identified risk factors can also inform risk stratification tools to support data-driven targeting of limited health resources.

Future research should explore the integration of longitudinal data to assess causal pathways and temporal dynamics between malaria exposure and anemia, validate the predictive models in country-specific and subnational contexts, and assess the feasibility of embedding machine learning–based risk prediction tools into routine health information systems. Expanding the models to incorporate clinical, nutritional, and environmental data may further enhance predictive accuracy and support more personalized and effective anemia prevention strategies.

## Supplementary Information

Additional file1

## Data Availability

The most recent data from the Demographic and Health Survey were used in this study, and it is publicly available online at (http:/www.dhsprogram.com). The datasets used and/or analyzed during the current study are available from the corresponding author on reasonable request.

## Abbreviations

EA: Enumeration area
LR: Logistic regression
ML: Machine learning
SBA: Skilled birth attendant
SDG: Sustainable Development Goal
SSA: Sub-Saharan Africa
