# Supplementary material for: An ensemble machine learning approach for predicting anemia among under-five children in malaria-endemic sub-Saharan African countries
Source: Infect Dis Poverty. 2026 Jul 13;15:78. doi: 10.1186/s40249-026-01461-6 (PMC13360475; doi:10.1186/s40249-026-01461-6)

**Appendix**

**Supplementary materials**

**Supplementary Figure 1**


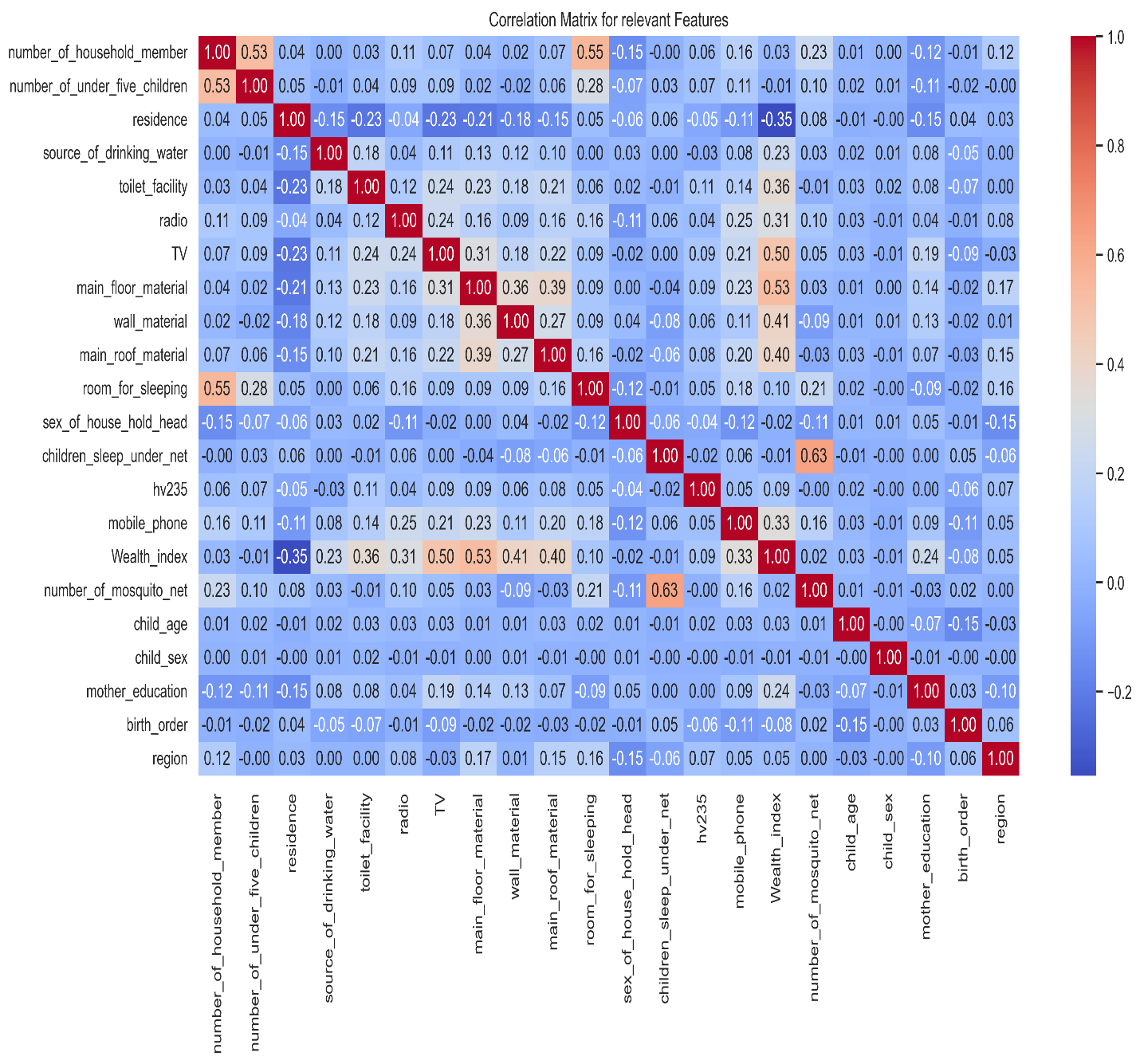


**Supplementary Figure 2**


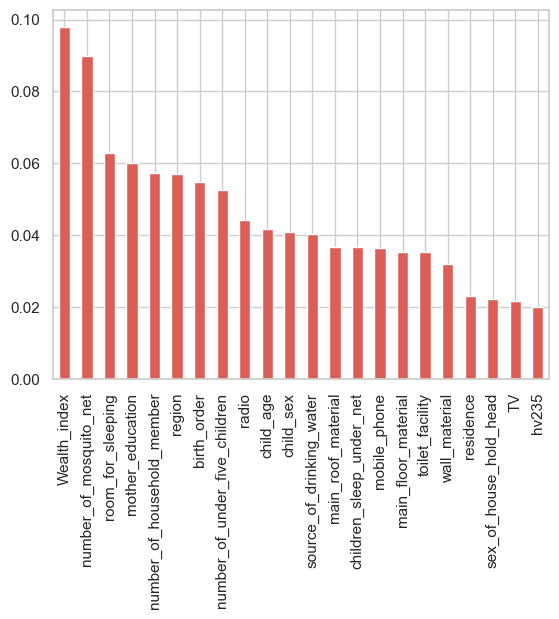


**Supplementary Figure 3**


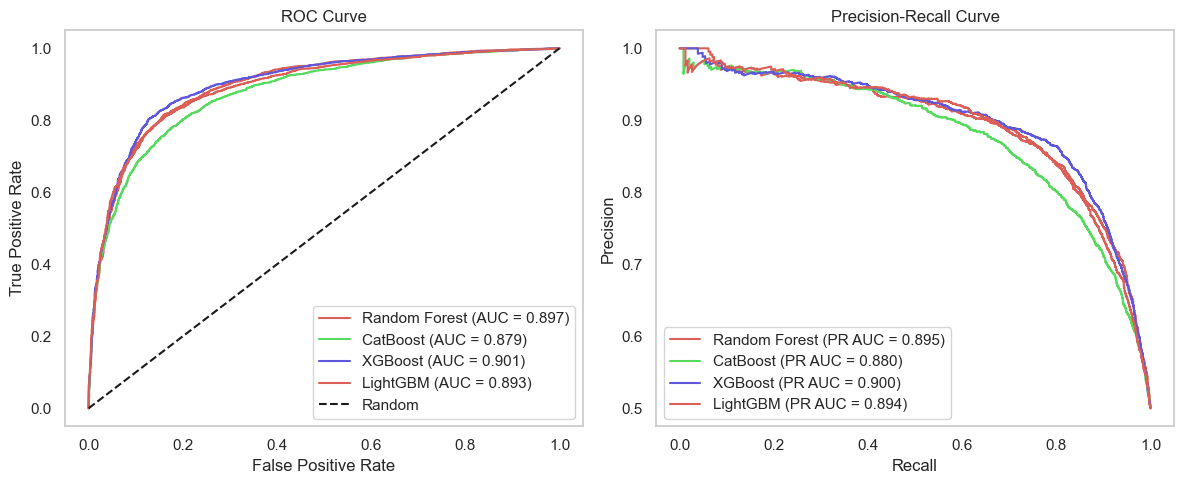


**Supplementary Figure 4**


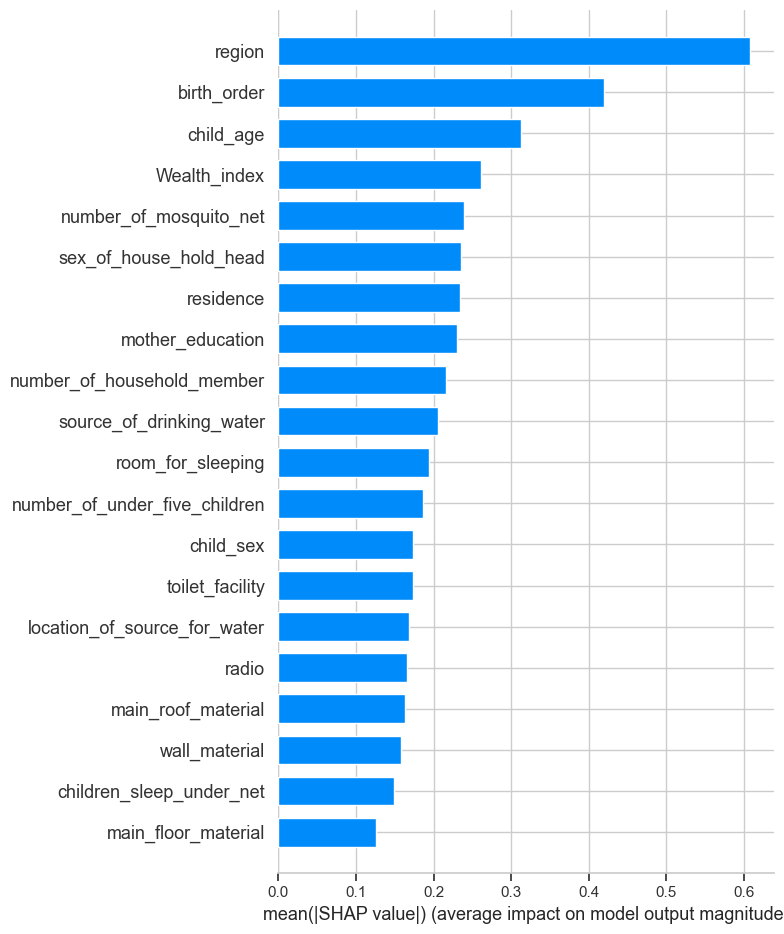


**Supplementary Figure 5**


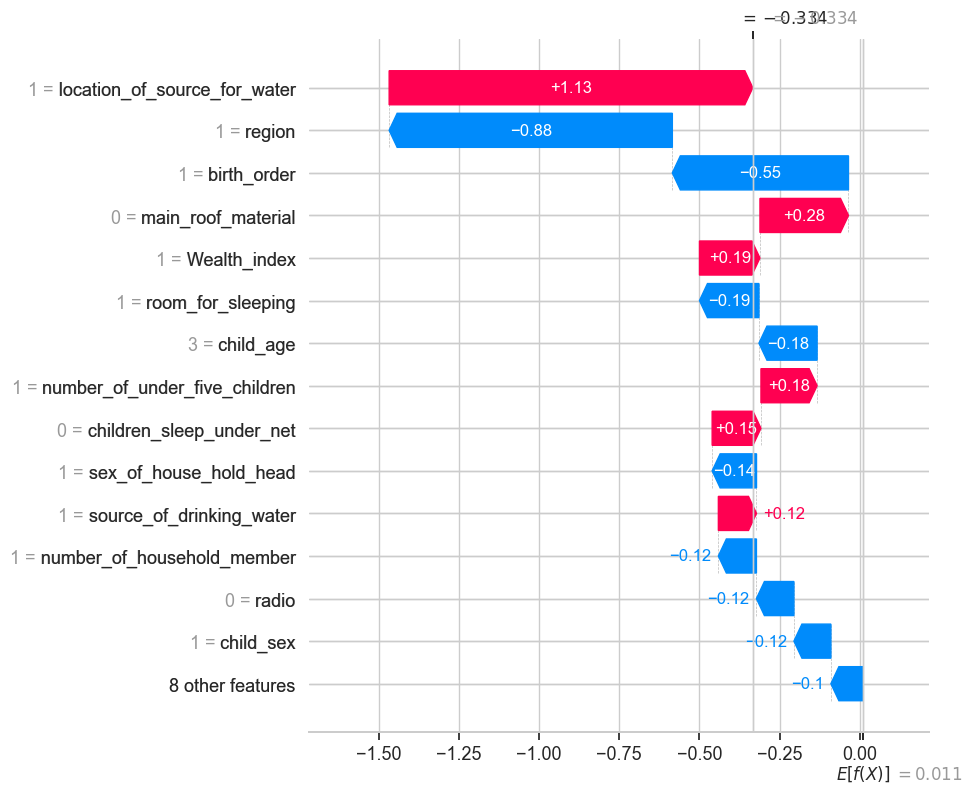

Supplement: Supplementary file 1 — Additional file1 [file 40249_2026_1461_MOESM1_ESM.docx]
